# Pregnancy in women living with perinatally acquired HIV: Perinatal outcomes and drug resistance profile

**DOI:** 10.1016/j.clinsp.2023.100174

**Published:** 2023-03-02

**Authors:** Gilmar de Souza Osmundo, Rafaela Alkmin da Costa, Rosa Maria Aveiro Ruocco, Rossana Pulcineli Vieira Francisco

**Affiliations:** a Disciplina de Obstetrícia, Departamento de Obstetrícia e Ginecologia, Faculdade de Medicina, Universidade de São Paulo (FMUSP), São Paulo, SP, Brazil; b Divisão de Clínica Obstétrica, Hospital das Clínicas, Faculdade de Medicina, Universidade de São Paulo (HCFMUSP), São Paulo, SP, Brazil

**Keywords:** HIV, Perinatology, Antiretroviral therapy, High-risk pregnancy, ART, Antiretroviral therapy, BHIVT, Behaviorally Acquired HIV, MTCT, Mother-To-Child Transmission, PHIV, Perinatally Acquired HIV, VL, Viral Load, WLH, Woman Living with HIV

## Abstract

**Objectives:**

To analyze the perinatal outcomes of Perinatally acquired HIV Infection (PHIV) in pregnant women.

**Method:**

This retrospective cohort study included singleton pregnancies in Women Living with HIV (WLH) between 2006 and 2019. Patient charts were revised, and maternal characteristics, type of HIV infection (perinatal vs. behavioral), Antiretroviral Therapy (ART) exposure, and obstetric and neonatal outcomes were assessed. The HIV-related aspects considered were: Viral Load (VL), CD4+ cell count, opportunistic infections, and genotype testing. Laboratory analyses were performed at baseline (first appointment) and 34 weeks of gestation.

**Results:**

There were 186 WLH pregnancies, and 54 (29%) patients had PHIV. Patients with PHIV were younger (*p* < 0.001), had less frequently stable partnerships (*p* < 0.001), had more commonly serodiscordant partners (*p* < 0.001), had a longer time on ART (*p* < 0.001), and had lower rates of undetectable VL at baseline (*p* = 0.046) and at 34 weeks of gestation (*p* < 0.001). No association was observed between PHIV and adverse perinatal outcomes. Among patients with PHIV, third trimester anemia was associated with preterm birth (*p* = 0.039). Genotype testing was available only for 11 patients with PHIV, who presented multiple mutations related to ART resistance.

**Conclusions:**

PHIV did not seem to increase the risk of adverse perinatal outcomes. However, PHIV pregnancies have a higher risk of viral suppression failure and exposure to complex ARTs.

## Highlights

• HIV viral resistance in pregnancy.
• Adverse perinatal outcomes in pregnant women living with HIV.
• Perinatally acquired HIV can increase the risk of viral suppression failure.

## Introduction

The number of people living with HIV has increased worldwide. Recent worldwide data estimated that 37.6 million persons were living with HIV, and there were 1.5 million new cases in 2020.^1^ In Brazil, pregnancy rates in Women Living with HIV (WLH) have increased by 22% in the past decade, with an incidence of 2.8 cases/1000 live births.^2^

Perinatally acquired HIV (PHIV) accounts for approximately 1% of HIV cases in Brazil.^2^ Advances in Antiretroviral Therapy (ART) have improved life expectancy and quality of life for this group of patients.^3^^,^^4^ Moreover, recent studies have demonstrated an increasing number of pregnancies in women living with PHIV.^3^^,^^5^

Available studies suggest that obstetric care of PHIV pregnant women may be challenging due to lower rates of viral suppression, higher rates of viral resistance, and exposure to complex, multidrug ART.^5^, ^6^, ^7^, ^8^, ^9^, ^10^ Furthermore, pregnant women present a higher prevalence of neurocognitive, psychiatric, and social issues.^3^^,^^5^^,^^10^

A few studies have assessed the outcomes of PHIV in pregnant women with controversial results on birth weight and preterm birth.^7^, ^8^, ^9^, ^10^, ^11^, ^12^, ^13^ This study aimed to analyze perinatal outcomes related to PHIV and possible risk factors.

## Materials and methods

This retrospective cohort study comprised singleton WLH pregnancies followed up at our obstetrics department (Hospital das Clínicas, São Paulo University Medical School, São Paulo – Brazil) between 2006 and 2019.

A database search was conducted to identify all cases of WLH evaluated in our department during the study period. Cases of multiple pregnancies, loss to follow-up, delivery at other hospitals, absence of prenatal care, unawareness of HIV diagnosis before delivery, and unavailability of hospital charts were not included.

Patient charts were revised, and data regarding maternal demographic characteristics, HIV infection, ART exposure, and obstetric and neonatal outcomes were assessed. HIV-related aspects considered were the type of transmission (perinatally vs. behaviorally), time of diagnosis, Viral Load (VL), CD4+ cell count, signs of immunodeficiency (CD4+ cell count < 200 mm^3^ or opportunistic infections), and genotype testing. Laboratory analyses were considered at baseline (first appointment) and 34 weeks of gestation.

Patients were considered to have PHIV if there was clear information on patients’ charts about such a type of transmission, based on HIV-positive mothers and diagnosis during childhood. Other cases were classified as Behaviorally acquired HIV (BHIV) cases.

The perinatal outcomes assessed included gestational age at delivery, preterm birth, low birth weight (< 2500 g), gestational diabetes, preeclampsia, fetal growth restriction, preterm labor, abnormal fetal well-being, and HIV Mother-To-Child Transmission (MTCT).

Throughout the study period, there were different guidelines for caring for pregnant WLH. Follow-up of this group of patients comprised regular prenatal appointments, first and second-trimester morphology scans, and regular assessment of fetal growth and wellbeing. All patients were prescribed ART, which consisted of three active antiretroviral drugs. The patients were assisted by a multidisciplinary team consisting of obstetricians, infectologists, psychologists, and social workers.

Historically, local practices regarding pregnant WLH tended toward elective cesarean delivery, irrespective of VL. Such policies have changed, and vaginal births have recently been encouraged. During the study period, zidovudine intrapartum prophylaxis and neonatal zidovudine syrup were routinely recommended to all the patients. Breastfeeding was contraindicated.

Indications for genotype testing during the study period included virological failure and, most recently, ART-*naïve* pregnant women. Data on genotype testing were assessed retrospectively, based on available test results on patients’ charts and the “Brazilian National Network of CD4+/CD8+ Lymphocyte Count and Viral Load” (SISCEL).

### Statistical analyses

Categorical data were presented as frequencies. Kolmogorov-Smirnov test and the values of kurtosis and skewness were employed to assess the distribution of numerical data. Variables presenting normal distribution were expressed as a mean and standard deviation; variables with non-normal distribution were expressed as median and interquartile range. Fisher's exact test and Chi-Square test were used to compare categorical variables. Student's *t*-test and Mann-Whitney *U* test were used to compare continuous variables presenting parametric and non-parametric distribution, respectively. McNemar's test was used to analyze paired nominal data (change in VL status). Unconditional logistic regression was applied to estimate Odds Ratios (ORs) and 95% Confidence Intervals (95% CIs). Statistical significance was set at p-value < 0.05. The data were analyzed using SPSS Statistics for Windows (version 20.0; IBM Corp., Armonk, NY, USA).

### Ethics

This study was in accordance with the Helsinki Declaration. The study was approved by the ethics committee of Hospital das Clínicas FMUSP, São Paulo, Brazil (CAAE 49,631,415.1.0000.0068, February 20, 2020).

## Results

During the study period, 241 WLH pregnancies were registered in our obstetrics department. Fifty-five pregnancies did not meet the inclusion criteria and therefore were excluded from the analysis. Sixteen patients had their prenatal follow-up elsewhere, 14 did not have any prenatal appointment, 10 had twin pregnancies, one delivered at another hospital, one patient had an HIV diagnosis at the time of delivery, and 13 cases’ hospital charts were unavailable. Thus, 186 WLH pregnancies were included, corresponding to 161 subjects: 24 women with two pregnancies during the study period and one with three pregnancies.

The demographic characteristics of the study population and prenatal, delivery, and neonatal information are presented in Table 1. The mean maternal age was 27.8 (± 7.7) years, and 77 (41.4%) women were nulliparous. Exactly 54 (29%) had PHIV. At the first appointment, 115 (61.8%) patients were already on ART, and the time of antiretroviral exposition was 4.0 (0–10) years. The most frequent antiretroviral drugs were Nucleoside-analog Reverse Transcriptase Inhibitors (NRTI) and Protease Inhibitors (PI) in 98.4% and 79% of the cases, respectively.

**Table 1:** Demographics, pregnancy, delivery, and neonatal characteristics of the 186 pregnant women living with HIV (WLH).

Parameters	Measure
Maternal age, years, mean (± SD)	27.8 (± 7.7)
GA at delivery, weeks	37.5 (± 1.8)
Birth weight, grams	2767.5 (± 556.3)
Nulliparous, n (%)	77 (41.4%)
HIV transmission	
Perinatally	54 (29%)
Behaviorally	132 (71%)
Undetectable Baseline VL	74 (39.8%)^1^
Undetectable VL at 34-weeks	108 (58.1%)
Mode of delivery	
- C-section	160 (95.2%)
- Vaginal	8 (4.8%)
Preterm birth	28 (16.7%)
Low birth weight	40 (24%)
MTCT	1 (0.6%)^2^

MTCT, Mother To Child Transmission; SD, Standard Deviation; VL, Viral Load.

1 missing information for 5 cases;

2 missing information for 12 cases.

PHIV and BHIV patients had major socio-demographic differences: Those with PHIV were younger (19.5 vs. 31.2 years, *p* < 0.001), had less frequent stable partnerships (38.5% vs. 74.2%, *p* < 0.001), and had more commonly serodiscordant partners (92.9% vs. 50.8%, *p* < 0.001) (Table 2). The PHIV group had a lower prevalence of systemic hypertension (none vs*.* 10.9%, *p* = 0.011) and smoking (11.1% vs. 24.2%, *p* = 0.011; Table 3).

**Table 2:** Clinical and socio-demographic features of pregnant women presenting perinatally acquired HIV (PHIV) and behaviorally acquired HIV (BHIV).

	PHIV (*n* = 54)	BHIV (*n* = 132)	
Variables	Number of Cases / Total available (%)	Number of Cases / Total available (%)	p
Maternal age, years, mean (± SD)	19.5 ± 2.9	31.2 ± 6.4	< 0.001^a^
Unplanned pregnancy	49 / 51 (96.1%)	112 / 128 (87.5%)	0.085^b^
Serodiscordant partners	26 / 28 (92.9%)	31 / 61 (50.8%)	< 0.001^b^
Stable partnership	20 / 52 (38.5%)	95 / 128 (74.2%)	< 0.001^b^
Smoking	6 /54 (11.1%)	31 / 128 (24.2%)	0.045^b^
Alcohol intake	1 / 54 (1.9%)	11 / 128 (8.6%)	0.113^b^
Recreational drugs	4 / 54 (7.4%)	22 / 128 (17.2%)	0.085^b^
Systemic Hypertension	0 (0%)	14 / 128 (10.9%)	0.011^b^
Baseline Anemia	15 / 50 (30%)	27 / 120 (22.5%)	0.302^b^
Baseline Lymphopenia	5 / 49 (10.2%)	6 / 117 (5.1%)	0.304^b^
Anemia 3rd trimester	23 / 50 (46%)	29 / 117 (24.8%)	0.007^b^
Lymphopenia 3rd trimester	2 / 50 (4.0%)	3 / 116 (2.6%)	0.638^b^

BMI, Body Mass Index; SD, Standard Deviation.

a Student's *t*-test.

b Fisher's exact test / Chi-square test.

**Table 3:** HIV-related features of pregnant women presenting perinatally acquired HIV (PHIV) and behaviorally acquired HIV (BHIV).

	PHIV (*n* = 54)	BHIV (*n* = 132)	
Variables	Number of Cases / Total available (%)	Number of Cases / Total available (%)	p
Time of HIV infection, years, mean (±SD)^1^	19.2 (± 3.1)	6.1 (± 5.2)	<0.001^a^
Time of ART, years, mean (±SD)^2^	14.2 (± 6.6)	3.5 (± 4.3)	<0.001^a^
ART			
- *PI*	51/54 (94.4%)	96/132 (72.7%)	0.001^b^
- *NRTI*	53/54 (98.1%)	130/132 (98.5%)	1.0^b^
- *NNRTI*	2/54 (3.7%)	31/132 (23.5%)	0.001^b^
- *Raltegravir*	13/54 (24.1%)	6/132 (4.5%)	<0.001^b^
- *Enfuvirtide*	1/54 (1.9%)	1/132 (0.8%)	0.497^b^
- *Maraviroc*	2/54 (3.7%)	0 (0%)	0.083^b^
*Previous OI*	11/50 (22%)	25/128 (19.5%)	0.712^b^
OI on pregnancy	5/54 (9.3%)	4/132 (3.0%)	0.124^b^
Undetectable baseline VL	15/50 (30%)	59/127 (46.5%)	0.046^b^
Undetectable VL at 34-weeks	23/51 (45.1%)	85/111 (76.6%)	<0.001^b^
Baseline CD4+ cells count, /mm^3^, median (IQR)^3^	441.5 (259.7–658.7)	440 (311.5–670)	0.691^c^
Baseline VL count, copies/mL, median (IQR)^4^	2968 (495–50,239)	3270.5 (633.5–23,211)	0.437^c^
CD4+ cells count at 34-weeks, /mm^3^, median (IQR)^5^	480 (243–662)	504.5 (332.2–703)	0.210^c^
VL count at 34-weeks, copies/mL, mean (±SD)^6^	3.1 (± 0.9)	2.8 (± 1.1)	0.237^c^

ART, Antiretroviral Therapy; IQR, Interquartile Range; NTRI, Nucleoside Analog Reverse Transcriptase Inhibitors; NNRTI, Non-Nucleoside Analog Reverse Transcriptase Inhibitors; OI, Opportunistic Infection; PI, Protease Inhibitors; SD, Standard Deviation; VL, Viral Load.

1 Data available for 170 cases.

2 Data available for 161 cases.

3 Data available for 175 cases.

4 Information available for 103 cases presenting detectable baseline viral load.

5 Data available for 161 cases.

6 Information available for 54 cases presenting detectable viral load at 34 weeks;.

a Student's *t*-test.

b Fisher's exact test / Chi-square test.

c ann-Whitney *U* test.

**Table 4:** Perinatal outcomes from the pregnancies of women presenting perinatally acquired HIV (PHIV) and behaviorally acquired HIV (BHIV).

	PHIV (*n* = 54)	BHIV (*n* = 132)			
Variables	Number of Cases / Total available (%)	Number of Cases / Total available (%)	p^a^	OR^b^	95% CI
Hospital admission	11/54 (20.4%)	21/132 (15.9%)	0.522	1.35	0.6–3.0
gestational diabetes	2/51 (3.9%)	9/121 (7.4%)	0.510	0.51	0.1–2.4
preeclampsia	2/51 (3.9%)	7/113 (5.8%)	0.726	0.7	0.1–3.3
Fetal growth restriction	8/51 (15.7%)	20/121 (16.5%)	1.0	0.9	0.4–2.3
Fetal loss	3/54 (5.6%)	13/132 (9.8%)	0.405	0.5	0.1–1.9
Preterm birth	7/51 (13.7%)	21/117 (17.9%)	0.499	0.7	0.3–1.8
Low birth weight	11/50 (22%)	29/117 (24.8%)	0.262	0.8	0.4–1.9
MTCT	0/49 (0%)	1/107 (0.9%)	1.0	‒	‒

MTCT, Mother-To-Child Transmission; OR, Odds Ratio.

a Chi-square test.

b Unconditional logistic regression.

PHIV patients had a longer time on ART (14.2 vs. 3.5 years, *p* < 0.001) and lesser rates of undetectable VL at the first prenatal appointment (30% vs. 40.5%, *p* = 0.046) and at 34 weeks of gestation (45.1% vs. 76.6%, *p* < 0.001). A change in the detectable baseline to undetectable VL at 34 weeks was observed in the BHIV group (*p* < 0.001) but failed to occur in the PHIV group (*p* = 0.267). However, the PHIV and BHIV groups had similar rates of baseline CD4+ cell count < 200 mm^3^ (22% vs. 16%, *p* = 0.348), opportunistic infections before pregnancy (22% vs. 19.5%, *p* = 0.712) and throughout gestation (9.3% vs. 3.0%, *p* = 0.124), and clinical hospital admissions (19.5% vs. 16%, *p* = 0.603).

ART regimens were markedly different among the groups. Compared with the BHIV group, PHIV patients’ ART comprised more frequently raltegravir (24.1% vs. 4.5%, *p* < 0.001) and PI (94.4% vs. 72.7%, *p* = 0.001), including darunavir (24.1% vs. 0.8%, *p* < 0.001). ART comprising more than 3 drugs was more common in the PHIV group (33.3% vs. 6.1%, *p* < 0.001). Non-Nucleoside Reverse Transcriptase Reverse Inhibitors (NNRTI) were more frequent in the BHIV group (*p* = 0.001).

Patients with PHIV had a higher rate of third-trimester anemia (46% vs. 24.8%, *p* = 0.007). In this subgroup of patients, third-trimester anemia was associated with detectable VL at 34 weeks (*p* = 0.001), baseline and 34-week CD4+ cell counts (*p* = 0.002 and *p* = 0.003, respectively), and trimethoprim-sulfamethoxazole intake (*p* < 0.001). There were no significant associations between third-trimester anemia and antiretroviral drugs (Supplementary Table 1).

Perinatal outcomes are shown in Table 4. No association was observed between PHIV and adverse perinatal outcomes. There was only one case of MTCT in a pregnant woman with BHIV infection. Among patients with PHIV, third-trimester anemia was associated with preterm birth (26.1% vs. 3.7%, *p* = 0.039) (Supplementary Table 2).

Genotype testing was available for only 11 pregnant women, and three cases did not present drug resistance. The genotype test results for patients with PHIV are summarized in Table 5. Patients with PHIV present with multiple mutations predominantly related to PI and NTRI resistance. Combined ART based on raltegravir and darunavir is the most frequent option for drug-resistant pregnant women.

**Table 5:** Genotype testing of pregnant women presenting perinatally acquired HIV (PHIV).

Patient	Age	CD4 (34 weeks)	VL (34 weeks) (log)	ART	PI	NRTI	NNRTI	Intermediate	Resistant
1	22	480	Undetected	DRV/R, RAL, AZT, 3TC, MVQ	L10V, K20T, L33F, M36I, M46I, I54V, T74P, V82A, L90M	K65R, M184V, 214F	V108I, Y181C	DRV, ETV	ATV, FPV, IDV, LPV, SQV, 3TC, ABC, EFV, NVP, TDF
2	20	134	426 (2.63)	TDF, 3TC, DRV/R	L10V, K20I, M36I, M46I, L63P, L90M	M184V, 214F	K103N, V179E, P225H	IDV, LPV, SQV, ABC	ATV, 3TC, EFV, NVP
3	21	991	Undetected	TDF, 3TC, ATV	‒	M184V, 211 K, 214F, T215S/Y	K101P, K103N/S	ABC, DDI	3TC, EFV, ETV, NVP
4	22	548	Undetected	3TC, RAL, DRV/R, MVQ	M36I, I54V, A71V, V82A, L90M	M41L, E44D, D67N, V75M, V118I, 211 K, 214F, T215Y, K219R	K101E, V106I, Y188L, G190A	3TC, ABC, DDI, ATV, FPV, IDV	AZT, EFV, TDF, ETV, NVP, SQV
5	21	105	837 (2.92)	TDF, 3TC, RAL, DRV/R, MVQ, T20	L10F, K20T, N88S	D67N, T215F, K219Q	V90I, K101P	IDV, ETV	ATV, NFV, AZT, EFV, NVP
6	20	322	176 (2.25)	TDF, 3TC, RAL, DRV/R	L10F, K20R, M36I, M46I, I47V, F53L, I54V, V82A	M41L, M184V, T215Y	‒	ABC, AZT, DDI, DRV	3TC, ATV, FPV, IDV, LPV, SQV
7	19	327	Undetected	TDF, 3TC, LPV/R, RAL	I13V, L63P, V77I	M184V, D67N, T69D, V75M, M184V, L210W, T215Y, K219Q	V108I, Y181C	ETV	3TC, ABC, AZT, DDI, EFV, NVP, TDF
8	19	131	4303 (3.63)	AZT, 3TC, NVP	M36I, I62V, L63P, V77I	M41L, D67N, T69D, V118I, L210W, T215Y	‒	3TC, ABC	AZT, d4T, DDI

ABC, Abacavir; ART, Antiretroviral Therapy; ATV, Atazanavir; AZT, Zidovudine; DDI, Didanosina; DRV, Darunavir; EFV, Efavirenz; ETV, Etravirine; FPV, Fosamprenavir; IDV, Indinavir; LPV, Lopinavir; MVQ, Maraviroc; NNRTI, Non- Nucleoside analogue Reverse Transcriptase Inhibitors; NRTI, Nucleoside Analogue Reverse Transcriptase Inhibitors; NVP, Nevirapine; PI, Protease Inhibitors; R, Ritonavir; RAL, Raltegravir; SQV, Saquinavir; TDF, Tenofovir; T20, Enfuvirtide; VL, Viral Load; 3TC, Lamivudine.

## Discussion

The present study is the largest single-center cohort study of PHIV pregnancies to date. Preterm birth and low birth weight were the most frequent events in the general study population (16.7% and 24%, respectively). Nevertheless, there was no association between the type of maternal HIV transmission and perinatal outcome.

Adverse perinatal outcomes have been described in previous small studies on PHIV pregnancies. Kenny et al. reported preterm birth in 14% of adolescent PHIV patients in the UK, a prevalence two times higher than that observed among teenagers in the same period of time.^14^ Williams et al. reviewed the records of PHIV pregnant teenagers and found that preterm delivery complicates 31% of pregnancies.^13^ Furthermore, a single-center cohort study of 14 pregnant WLH showed an increased risk for small for gestational age newborns in PHIV pregnancies.^11^ Retrospective studies also suggest a lower birth weight in PHIV pregnancies than in BHIV cases.^15^^,^^16^

Conversely, Lazenby et al. recently assessed a multicentric cohort of 41 PHIV gestations and found higher birth weight in the PHIV group, in addition to similar rates of live birth, low birth weight, MTCT, and neonatal intensive care admissions between the PHIV and BHIV groups.^7^

The present findings suggest that PHIV does not increase the odds of preterm birth, low birth weight, fetal growth restriction, fetal loss, or maternal risks of preeclampsia, gestational diabetes, and hospital admission. These findings are consistent with those of other recent retrospective case-control studies on PHIV pregnancies.^8^^,^^12^^,^^17^ Badell et al. retrospectively assessed 20 cases of PHIV in pregnant women and found no association between the type of HIV transmission and major obstetric complications.^17^ Jao et al. reported similar rates of preterm birth and low birth weight in the PHIV group after analyzing 2692 pregnant WLH (270 cases of PHIV) from two North-American cohort studies.^12^

Compared with BHIV patients, the PHIV population had a higher prevalence of third-trimester anemia, which was associated with an increased risk of preterm birth. Anemia has been described as a frequent complication of WLH pregnancies, ranging from 37% to 64%;^18^, ^19^, ^20^, ^21^ thus, the authors observed an even lower anemia rate in the present study population. A large retrospective Brazilian cohort study found anemia in 56% of pregnant women with WLH, which was associated with low CD4+ cell count and did not have any association with ART.^20^ Williams et al. reported 50% anemia in a small study of PHIV pregnancies;^13^ however, data on anemia in pregnant women with PHIV are still scarce. Although the present findings suggest that anemia should be screened and promptly treated in pregnant women with PHIV due to the risk of preterm delivery, the authors had only a small number of anemia cases, and this study was not designed to evaluate preterm birth risk factors. Moreover, despite anemia being more frequent among PHIV patients, the authors did not find an increased risk of preterm births in this group of patients. Larger multicenter studies are necessary to properly analyze the risk factors for preterm birth in pregnant women with PHIV.

The management of ART seemed to be more complex in our PHIV group than in the BHIV group. PHIV pregnancies were more frequently exposed to antiretroviral drugs, ART combinations comprising >3 drugs, and alternative drugs, such as raltegravir and darunavir. Currently, the Brazilian HIV guidelines consider raltegravir-based ART as the first option for pregnant women;^22^ however, during the study period, the first option to treat pregnant WLH included a combination of two NRTI plus one NNRTI or PIs (lopinavir or atazanavir/ritonavir).^20^ Despite the complexity of ART, patients with PHIV presented more frequently with detectable VL at the first appointment and at 34 weeks of gestation. Moreover, patients with PHIV presenting with detectable baseline VL achieved viral suppression less frequently in the third trimester.

Although there were higher rates of detectable VL and failure to suppress viral infection among our PHIV patients, this group presented neither an increased risk of MTCT nor a higher prevalence of immunodeficiency signs, such as opportunistic infections and CD4+ cell count <200 mm^3^.

ART in PHIV patients tends to be more challenging since these patients have longer periods on ART and multiple expositions to antiretroviral drugs.^9^^,^^12^^,^^23^ Previous studies have reported lower CD4+ cell counts and higher VL in pregnant women with PHIV.^8^, ^9^, ^10^^,^^16^^,^^23^ Byrne et al. assessed WLH pregnancies in the UK and Ireland and reported that the risk of detectable VL near delivery was three times higher in PHIV patients.^10^ Lundberg et al. analyzed a cohort of 32 young Brazilian PHIV pregnant women and found that only 32% of the patients had baseline VL < 1000 copies/mm^3^; however, they found similar baseline CD4+ cell counts and VL in PHIV and BHIV pregnancies.

Low ART adherence is a plausible cause of reduced viral suppression in PHIV patients.^3^^,^^10^^,^^24^ Studies on young patients with PHIV have shown that ART adherence depends on how individuals cope with personal and structural barriers,^3^^,^^25^^,^^26^ such as issues related to health insurance, employment, education, and problems dealing with family and taking care of children.^25^ Regarding adherence during pregnancy, Trahan et al. reported that 45% of pregnant women with PHIV self-reported poor ART adherence in a Canadian cohort study.^27^

In this regard, the lack of ART adherence and subsequent lack of viral suppression among PHIV patients might be related to the socio-demographic aspects of such a population. In the present findings, PHIV pregnant women were younger, had fewer stable partnerships, and more commonly had serodiscordant partners. Although not statistically significant, pregnancies were unplanned in 96% of patients with PHIV. Colombini et al. reviewed studies on ART adherence during pregnancy and suggested that HIV-related stigma, fear of disclosure of HIV diagnosis, young maternal age, low income, and lack of partnership may be associated with low ART adherence.^28^ Brittain et al. performed a large cross-sectional study on pregnant WLH and reported that single marital status and unplanned pregnancy were associated with suboptimal adherence and elevated VL, respectively.^29^

Another plausible reason for lower viral suppression in patients with PHIV is viral resistance to ART. Although genotype testing was available only for a small sample of our PHIV population, multiple antiretroviral resistance mutations were confirmed in 73% of the tested cases, and five patients had multiclass resistance to NRTI, NNRTI, and PI. The present results might have a selection bias since genotype testing was only performed in cases with virological failure and risk of viral resistance. Nonetheless, previous studies reported an association between PHIV and ART resistance. Teixeira et al. analyzed a Brazilian cohort of 232 pregnant WLH and found that 15% of the population had at least one antiretroviral resistance.^30^ Lazenby et al. performed a multicenter case-control study on antiretroviral resistance and pregnancy and reported an association between PHIV infection and antiretroviral resistance.^7^

## Conclusions

In conclusion, PHIV did not seem to increase the risk of adverse perinatal outcomes. However, PHIV pregnancies have a higher risk of viral suppression failure and exposure to complex ART. Such patients should be followed up by an experienced multidisciplinary team familiar with the management of viral resistance. Social issues regarding PHIV pregnancies should also be considered to reduce risks and vulnerability. Larger and multicenter studies are necessary to properly address PHIV-related perinatal risk.

## Authors' contributions

Osmundo Junior GS was responsible for study conception and design, data collection, analysis, interpretation, and manuscript drafting. Costa RA participated in the analysis and revision of the manuscript for intellectual content. Ruocco RMA and Francisco RPV reviewed the manuscript and contributed to relevant discussions. All the authors approved the final version of the manuscript.

## Funding

This research did not receive any specific grant from funding agencies in the public, commercial, or not-for-profit sectors

## Conflicts of interest

The authors declare no conflicts of interest.

## Appendix Supplementary materials

Image, application 1
