# Supplementary material for: Pregnancy in women living with perinatally acquired HIV: Perinatal outcomes and drug resistance profile
Source: Clinics (Sao Paulo). 2023 Mar 2;78:100174. doi: 10.1016/j.clinsp.2023.100174 (PMC9995930; doi:10.1016/j.clinsp.2023.100174)
Supplement: Supplementary file 1 [file mmc1.docx]

**CLINICS-D-22-00598_Supplementary Material**

**Supplementary Table 1** Association between anemia and clinical features of pregnant women presenting Perinatally acquired HIV (PHIV).

|  | **Anemia** | |  |
| --- | --- | --- | --- |
|  | **Yes (23)** | **No (27)** |  |
| **Variables, n (%)** | **Cases / Total available (%)** | **Cases / Total available (%)** | **p** |
| Maternal age, years, mean (± SD) | 19.4 ± 2.1 | 19.4 ± 3.1 | 0.898**^a^** |
| Time of HIV infection^1^, years, mean (± SD) | 19.5 (± 2.0) | 18.9 (± 3.3) | 0.306**^a^** |
| Time of ART^2^, years, mean (± SD) | 13.9 (± 5.9) | 13.7 (± 6.9) | 0.641**^a^** |
| BMI, kg/m^2^, median (IQR)^1^ | 22.9 (19.4–25.2) | 22.4 (21.3–25.8) | 0.366**^b^** |
| Previous OI | 6 / 20(30%) | 4 / 26 (15.4%) | 0.292**^c^** |
| OI on pregnancy | 5 / 23 (21.7%) | 0 / 27 (0%) | 0.016**^c^** |
| ART |  |  |  |
| *PI* | 20 / 23 (87%) | 27 / 27 (100%) | 0.090**^c^** |
| *NRTI* | 23 / 23 (100%) | 27 / 27 (100%) | ‒ |
| *NNRTI* | 2 / 23 (8.7%) | 0 / 27 (0%) | 0.207**^c^** |
| *Raltegravir* | 5 / 23 (21.7%) | 8 / 27 (29.6%) | 0.526**^c^** |
| *Enfuvirtide* | 1 / 23 (4.3%) | 0 / 27 (0%) | 0.460**^c^** |
| *Maraviroc* | 0 / 23 (0%) | 2 / 27 (7.4%) | 0.493**^c^** |
| Baseline CD4+ cells count < 200 mm^3^ | 9 / 21 (42.9%) | 1 / 25 (4.0%) | 0.003**^c^** |
| Undetectable baseline VL | 5 / 21 (23.8%) | 10 / 25 (40.0%) | 0.243**^c^** |
| CD4+ cells count < 200 mm^3^ at 34 weeks | 12 / 23 (52.2%) | 0 / 27 (0%) | <0.001**^c^** |
| Undetectable VL at 34 weeks | 4 / 23 (17.4%) | 18 / 27 (66.7%) | <0.001**^c^** |
| TMP-SMX | 11 / 23 (47.8%) | 1 / 27 (3.7%) | <0.001**^c^** |

ART, Antiretroviral Therapy; BMI, Body Mass Index; IQR, Interquartile Range; NTRI, Nucleoside analog Reverse Transcriptase Inhibitors; NNRTI, Non-Nucleoside analog Reverse Transcriptase Inhibitors; PHIV, Perinatally acquired HIV; PI, Protease inhibitors; SD, Standard Deviation; TMP-SMX, Trimethropim and Sulfamethoxazole.

^1^ Data available for 50 cases; ^2^ Data available for 36 cases.

^a^ Student’s *t*-test; ^b^ Mann-Whitney *U* test; Chi-square / Fisher’s exact test.

**Supplementary Table 2** Association between anemia and perinatal outcomes on pregnancies from women presenting perinatally acquired HIV (PHIV).

|  | **Anemia** | | **p^a^** |
| --- | --- | --- | --- |
|  | **Yes (23)** | **No (27)** |  |
| **Variables, n (%)** | **Cases / Total available (%)** | **Cases / Total available (%)** |  |
| Hospital admission | 7 / 23 (30.4%) | 4 / 27 (11.8%) | 0.184 |
| Gestational diabetes | 0 / 23 (0%) | 2 / 27 (7.4%) | 0.493 |
| Pre-eclampsia | 0 / 23 (0%) | 2 / 27 (7.4%) | 0.493 |
| Fetal growth restriction | 4 / 23 (17.4%) | 4 / 27 (14.8%) | 1.0 |
| Prematurity | 6 / 23 (26.1%) | 1 / 27 (3.7%) | 0.039 |
| Low birth weight | 6 / 22 (27.3%) | 4 / 27 (14.8%) | 0.311 |

^a^ Fisher’s exact test / Chi-square test.
